# Unexpected deaths of children and young people in the UK

**DOI:** 10.1192/pb.bp.114.049825

**Published:** 2016-04

**Authors:** Paul Stallard, Michelle Maguire, Justin Daddow, Rosie Shepperd, Mike Foster, Jill Berry

**Affiliations:** 1 University of Bath; 2 Oxford Health NHS Foundation Trust

## Abstract

**Aims and method** To review the deaths of children and young people who took their own life. We conducted a retrospective analysis of serious incident reports from a National Health Service trust and reviews by the child death overview panels of the local safeguarding children boards.

**Results** We identified 23 deaths, with annual rates varying considerably between local authorities and over time. Over half of the children (*n* = 13, 56%) were not known to specialist child and adolescent mental health services, with 11 having no contact with any agency at the time of their death. Hanging was the most common method (*n* = 20, 87%) and of these, half (*n* = 11, 55%) were low-level hangings.

**Clinical implications** Training is required to improve awareness, recognition and the assessment of children at risk of taking their own life. Specialist child mental health services should directly assess plans or attempts at hanging and offer advice about the seriousness of attempting this. National data (by age) on children and young people who take their own life should be routinely published to inform clinical and preventive services.

In 2009, suicide was the leading cause of death in people aged 5-34 years in England and Wales.^1^ The reduction of death by suicide is an important public health objective,^2^ although few studies have reported rates of suicide in young people under the age of 18.^3,4^ Establishing a profile of children who take their own life is difficult since there is no single contemporary database. The UK national suicide database does not include children younger than 15 and there is a 2-year delay in reporting statistics, which hinders the identification of current trends.^5^ The National Confidential Inquiry into Suicide and Homicide by People with Mental Illness includes children older than 10 but only those known to child mental health services at the time of their death.^6^ Local safeguarding children boards (LSCBs) have a statutory responsibility to review the deaths of all children up to the age of 18 normally resident within their area.^7^ However, published data relate to reviews completed within each calendar year rather than actual deaths each year.^8^ For the year ending 31 March 2014 a total of 28% of reviews were not completed within 12 months.

Establishing a contemporary profile of children who take their own life can alert providers to current fatal trends, target vulnerable groups and proactively inform local safeguarding training. However, in addition to an absence of cohesive and contemporary data, establishing a comprehensive picture is also compounded by grouping and definitional issues. Suicide rates significantly increase during adolescence but are currently reported within wider age bands rather than specific age groups.^4,5^ Furthermore, child mental health services typically go up to the age of 18 and yet the current reporting by the Office for National Statistics is of suicides in 15- to 19-year-olds. Establishing a comprehensive picture is further complicated by the caution in reaching suicide verdicts in children and the increasing use of narrative verdicts.^9,10^ Narrative verdicts summarise the nature and circumstances of the death where intent is difficult to determine, a factor that may be particularly relevant for young people and one that will result in suicide rates being underreported.

Given these limitations, a recent analysis of suicide and accidental deaths in children aged 10-19 years in England and Wales between 2001 and 2010 identified a total of 1523 deaths.^4^ Males were more likely than females to take their own lives, with hanging being the most common method. Rates in 15- to 19-year-old males fell over the 10-year period but in the younger group low numbers prevented a more thorough investigation of trends.

In view of the absence of national contemporary and comprehensive data a local review was conducted of unexpected deaths in children and young people under the age of 18 residing within five local authorities.

## Method

We conducted a retrospective case analysis of unexpected deaths by possible suicide of young people under the age of 18 during the period from 1 April 2010 to 31 December 2013. Young people who lived within the geographical area of a trust delivering mental health services spanning five local or unitary authorities were included.

The National Patient Safety Agency has developed a framework for the identification and reporting of serious incidents in the National Health Service.^11^ Serious incident reports undertaken by the trust were reviewed for all cases known to the child and adolescent mental health services (CAMHS) during the review period. Each of the LCSBs responsible for the five local or unitary authorities was contacted to request reports on young people not known to CAMHS who took their own life. Support was obtained from all LCSBs and information from the local child death overview panels was reviewed. Records were systematically analysed and demographic data, method of death, contributing and risk factors were extracted.

## Results

A total of 24 young people aged 10-17 were identified by the review. In one instance the child death overview panel did not find any evidence to suggest that the young person intended to take their own life (overdose). This case was removed from the analysis.

### Demographic profile

Table 1 provides a profile of the children in each of the geographical areas included in this audit with the English national average.

**Table 1:** Population, ethnicity, poverty, looked after children, academic attainments and hospital admissions following self-harm

	LA1	LA2	LA3	LA4	LA5	England
Total population, *n*	653 798	505 283	176 016	470 981	209 156	53 012 456

Children aged 0–17 years, %	21.1	22.9	19.1	22.0	22.1	21.4

School population (aged 5–16) from Black and minority ethnic groups, %	18.3	25.9	9.5	8.2	19.0	25.6

Children under 16 in poverty,^a^ %	12.7	10.8	13.5	12.1	17.8	21.1

Children in care^b^	33.0	32.0	49.0	40.0	54.0	59.0

Good academic performance,^c^ %	57.9	69.7	57.5	59.3	52.7	59.4

Hospital admissions for self-harm^d^	118.1	68.4	133.5	126.7	176.3	115.5

LA, local authority.

a. Children aged under 16 living in families in receipt of out-of-work benefits or tax credits and with reported income of less than 60% median income, 2010.

b. Rate of children looked after at 31 March per 10 000 population aged under 18, 2012.

c. Pupils achieving five or more GCSEs or equivalent including maths and English, 2011/12.

d. Crude rate per 100 000 (age 0–17 years), 2011/12.

Compared with the English national average, there were fewer children from Black and minority ethnic groups living in poverty and looked after by social care. In all but one area, rates of hospital admission following self-harm were higher.

### Suicide rates by geographical area

Figures from the UK national census identified a total population of 2 048 832 residing within the geographical area of this review.^12^ Of these, 437 131 (21.3%) were under the age of 18 with 197407 (9.6%) aged 10-17 (Table 2).

**Table 2:** Population and suicide by area

	LA1	LA2	LA3	LA4	LA5
Age group, years: *n*					
0–4	41 056	31 832	9238	28 373	14 083
5–9	36 031	30 939	8700	27 199	12 273
10–14	37 335	32 552	9568	29 276	12 433
15–17	23 539	20 222	6196	18 552	7734
Total 0–17	137 961	115 545	33 702	103 400	46 523
Total 10–17	60 874	52 774	15 764	47 828	20 167

Deaths, *n*					
Apr–Dec 2010	2	0	0	3	0
Jan–Dec 2011	2	1	2	3	0
Jan–Dec 2012	3	2	2	0	0
Jan–Dec 2013	2	0	1	0	0
Total	9	3	5	6	0

Deaths, annual rate per 100 000	3.94	1.52	8.46	3.34	0

LA, local authority.

Rates of suicide per 100 000 population were calculated (i.e. total number of deaths×population/100 000÷3.75 years), giving an annual rate of 3.11 per 100 000 population aged 10-17. Assuming the same population base for each year there was considerable variability between years (i.e. 1.90 in 2010, 4.05 in 2011, 3.55 in 2012, 1.52 in 2013 per 100 000). Similarly, there were large differences between local authorities, with one area having no unexpected deaths during the review, whereas another had 9 (Table 2).

### Profile

Of the 23 children, 10 (44%) were known to specialist child mental health services, 2 were looked after, 6 were known to youth offending services or the police and 6 had identified school problems. Eleven children had no contact with health, education, social care or youth justice services at the time of their death. Consistent with English national trends, about two-thirds were male and aged ⩾15 (Table 3).

**Table 3:** Characteristics of children known and not known to specialist child and adolescent mental health services (CAMHS)

	Known to CAMHS, *n* = 10	Not known CAMHS, *n* = 13
Gender, *n*		
Male	5	9
Female	5	4

Age at death, years		
11	0	1
12	0	0
13	2	3
14	0	1
15	2	2
16	2	3
17	4	3

Ethnicity, *n*		
British White	8	11
Other	2	2

Year of death		
2010	2	3
2011	2	6
2102	4	3
2013	2	1

Month of death		
Winter (January–March)	2	3
Spring (April–June)	1	5
Summer (July–September)	4	4
Autumn (October–December)	3	1

Day of death		
Monday	2	1
Tuesday	1	4
Wednesday	2	0
Thursday	0	2
Friday	1	3
Saturday	4	3
Sunday	0	0

Time of death		
Morning	1	1
Afternoon	5	4
Evening/night	4	7
Unknown	0	1

Location		
Home and garden	9	8
Public place	1	5

There was an increase in the number of young people who took their own life in 2011, which is consistent with national figures.^1^ Deaths were spread across the year with the largest number occurring in March and July (four each). Almost half occurred on a Friday or Saturday and during the evening/night. No deaths occurred on a Sunday or in December or June. The majority of deaths happened at home or in the garden.

### Circumstances surrounding the death

The most common cause of death was hanging (*n* = 20), with the remaining deaths resulting from shooting (*n* = 2) and jumping (*n* = 1). Of those who died from hanging, in case of 11 children these were low-level hangings (e.g. door handles, window catches), 8 were high-level suspensions and in one case it was unclear.

Evidence of suicide notes, texts or farewell messages left on computers were found in 10 cases and of these, 7 children had not been known to CAMHS. There were a further two incidents (both known to CAMHS) where a young man had previously posted threats to kill himself and a young woman stated that she had planned her death.

It proved extremely difficult to obtain detailed information about events that may have precipitated the death and in many instances no immediate or clear trigger could be identified. Where it could be identified death occurred after fairly routine everyday events such as failing a driving test, minor family or sibling argument, discussion about poor school grades, bad day at school. In other instances there were less immediate but potentially important events that might have contributed to the young person's decision to take their own life such as breaking up with a girlfriend, drug use and a recent court appearance.

### Potential risk factors

We searched for information on a number of potential risk factors such as previous self-harm, drug use, sexual identity, internet use, peer relationships/bullying. Recording of this information in the documents examined was poor.

A known history of previous self-harm was reported in 8 children of whom 5 had made previous serious suicide attempts including an overdose (*n* = 1) and past hanging/ligature tying (*n* = 4). All children with a known history of self-harm had been involved with CAMHS; 3 had previously been in-patients with 6 being open cases to CAMHS at the time of their death.

In 6 cases (5 known to CAMHS) significant alcohol and/or drug misuse was identified. Although no sexual identity issues were identified, there were concerns about the young person's sexual behaviour in 6 cases (all known to CAMHS). These included significant past sexual abuse, involvement in prostitution, relationship with a significantly older partner and an extreme reaction to witnessing the sexual behaviour of a parent.

Information about internet usage was poorly documented, although in four more recent cases there was evidence that young people had been researching internet sites for information about how to carry out a suicide.

In terms of peer relationships, being the victim of current bullying was not identified in any case. Although some young people had a limited social circle, significant friendship issues that may have contributed to their decision to take their own life were identified in only one case. In this instance a young person's relationship ending with their significantly older partner appears to have influenced their decision.

## Discussion

Before discussing the implications of this review, its limitations need to be acknowledged. First, there was considerable variability in the quality and comprehensiveness of the reports examined. Some of the variables we investigated may have been present but were not identified or fully detailed in the information reviewed. Second, our sample is small which can result in trends being under- or overinflated or skewed by a few cases. Third, it was difficult to ascertain whether issues were historical or current and whether they were directly related to the young person's death. Fourth, this review spanned almost 4 years during which awareness of potentially important factors such as social media has increased. These factors may have been present in earlier cases but may not have been investigated.

Our review demonstrates that it is challenging to gain a comprehensive and contemporary understanding of young people who take their own life. From a service perspective it is difficult to determine whether variations in rates over time are indicative of a growing national problem or reflect a worrying local trend. The local increase observed in 2011, which led to this review, was ultimately shown to reflect a national increase during that year. However, even within this annual rise there was considerable variability across the five local authorities that participated in this review.

Similarly, although numbers are small, detailing specific ages rather than age bands has highlighted the deaths of a number of 13-year-olds. Detailing these has facilitated useful discussions with LCSBs and informed suicide prevention strategies and the specific age group to target. We therefore endorse the recommendation from Windfuhr and colleagues^4^ to routinely publish statistics on suicides in children aged 10 and above. We would however advocate that this should be done by specific age rather than by age band groupings since this may more helpfully inform suicide prevention strategies.

Our review has highlighted a number of issues which merit particular attention. First, the majority of children who took their own life were not known to specialist CAMHS, with almost half having no contact with any service at the time of their death. Keeping children safe is not the responsibility of a single service or agency. Everyone who has contact with children and young people has a role and training may be required to improve awareness, recognition and assessment of at-risk children. Second, hanging was by far the most common method used by children to take their own life. Risk assessments should therefore routinely and directly enquire about thoughts, research (e.g. internet websites), plans or previous attempts at hanging. Specialist CAMHS should also consider the advice they provide to young people who are considering or have experimented with ligatures. These are high-risk behaviours and their potential seriousness should be directly discussed with the young person. This was strongly endorsed by a young person user group who felt that many were not fully aware of the seriousness or speed of death that could result from ligatures. Finally, these findings have implications for the out-of-hours support provided by specialist CAMHS for young people who are at risk of taking their own life. Targeting support on Friday and Saturday evenings, the times when deaths were more likely to occur, might be helpful.

Our review has demonstrated how difficult it is for local services to gain contemporary information about young people who take their own life. Reporting deaths by age and the adoption of a standardised framework for assessing risk and contributing factors would be a useful first step to increase our understanding.
